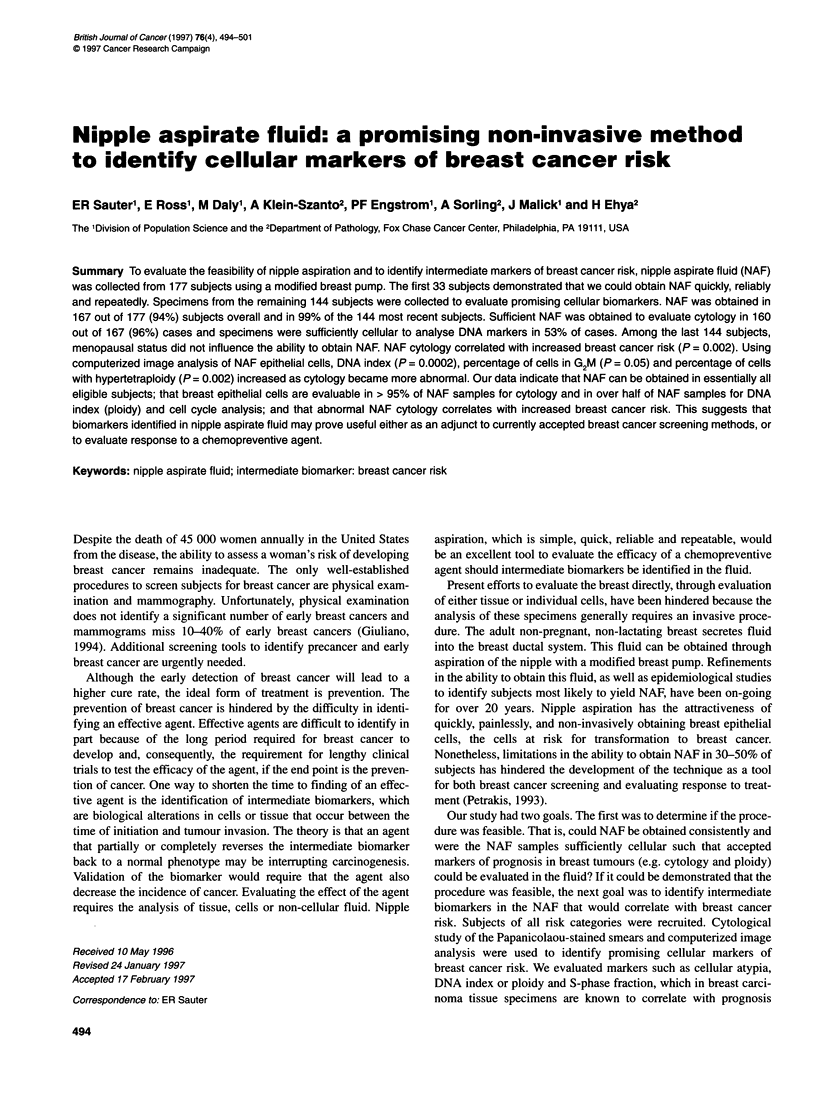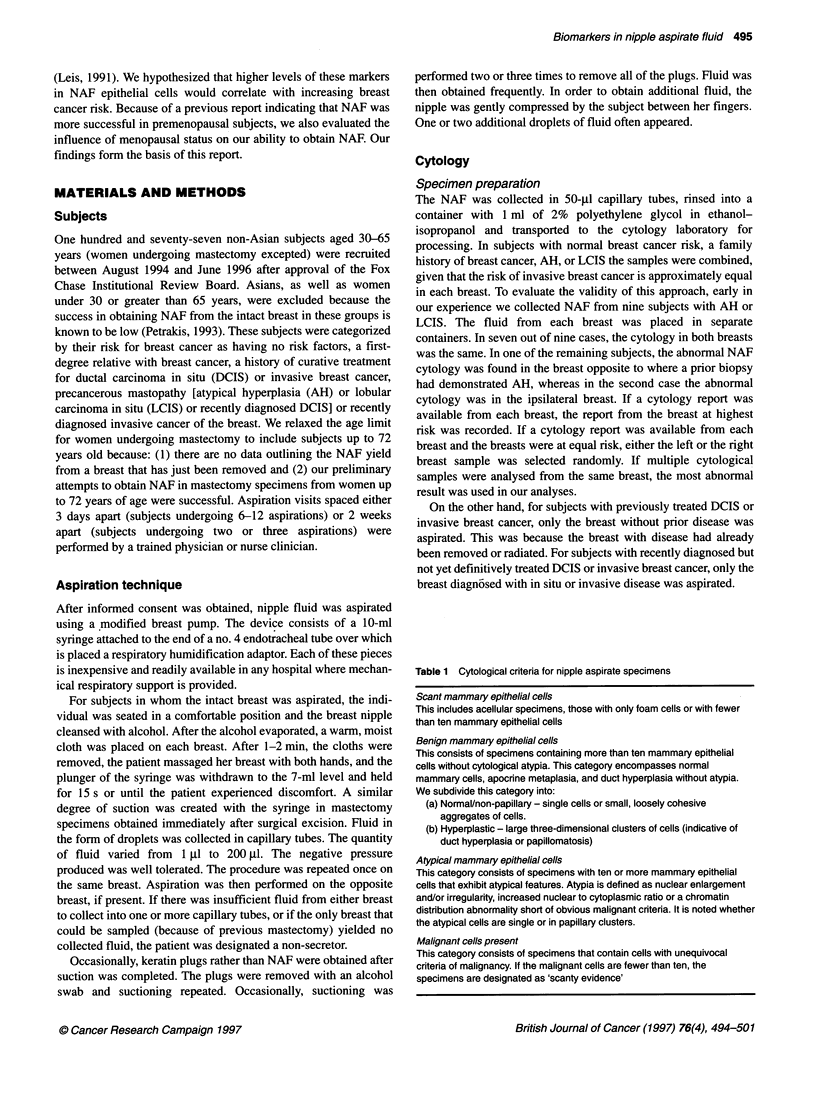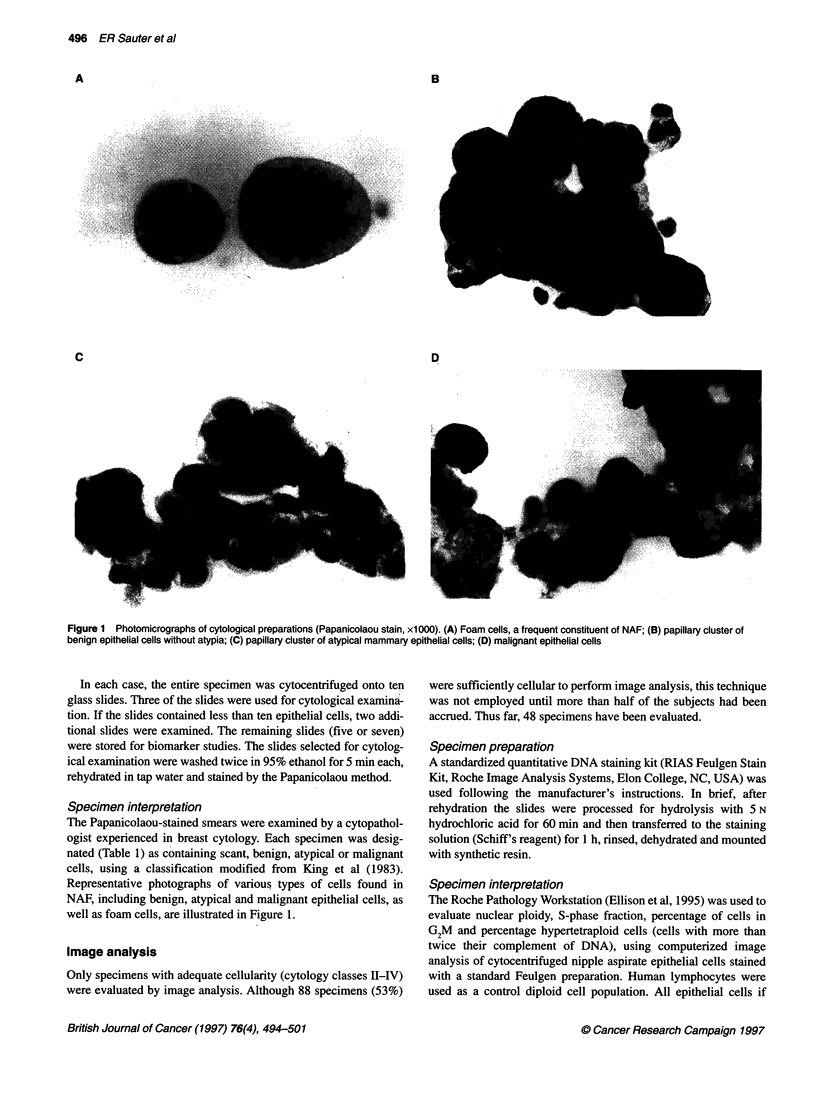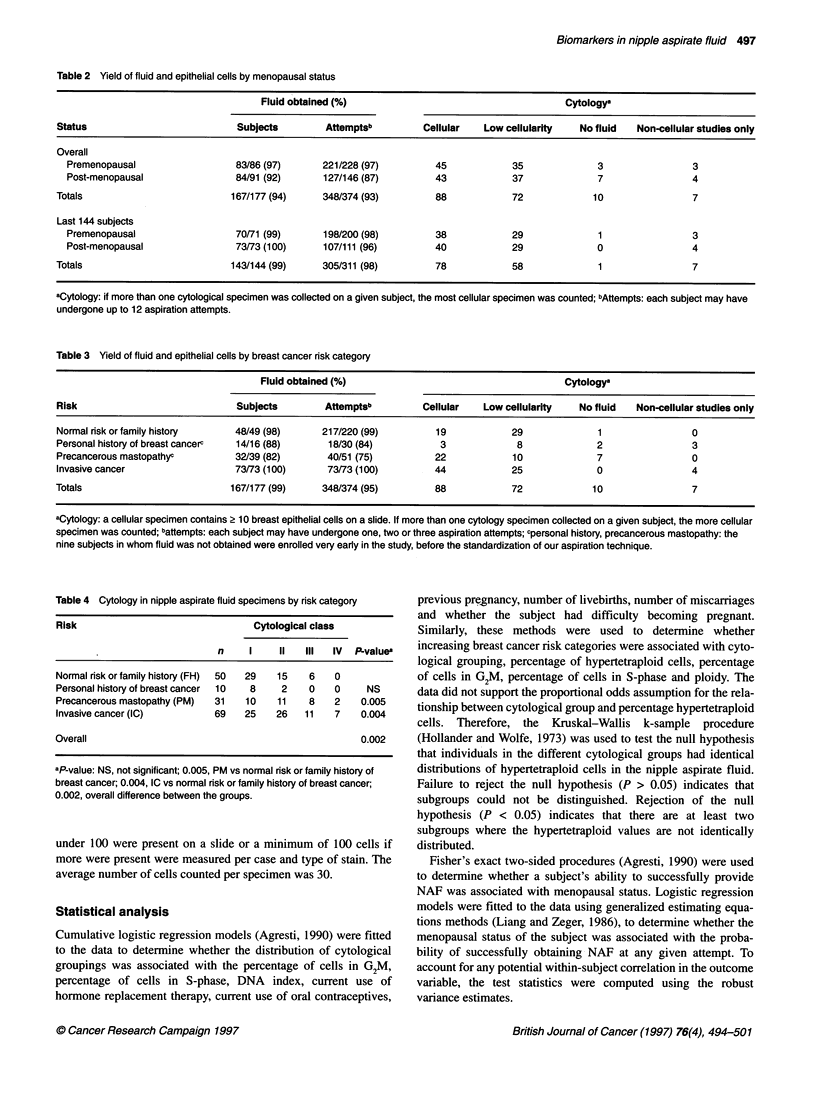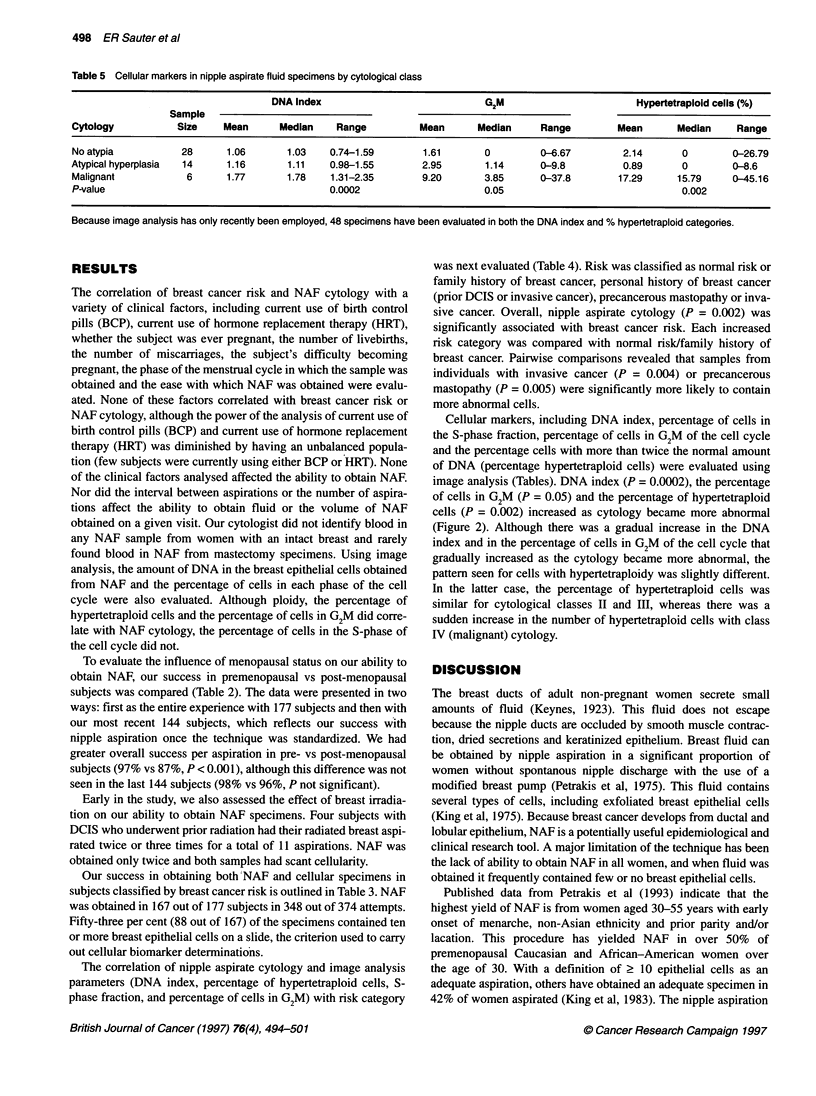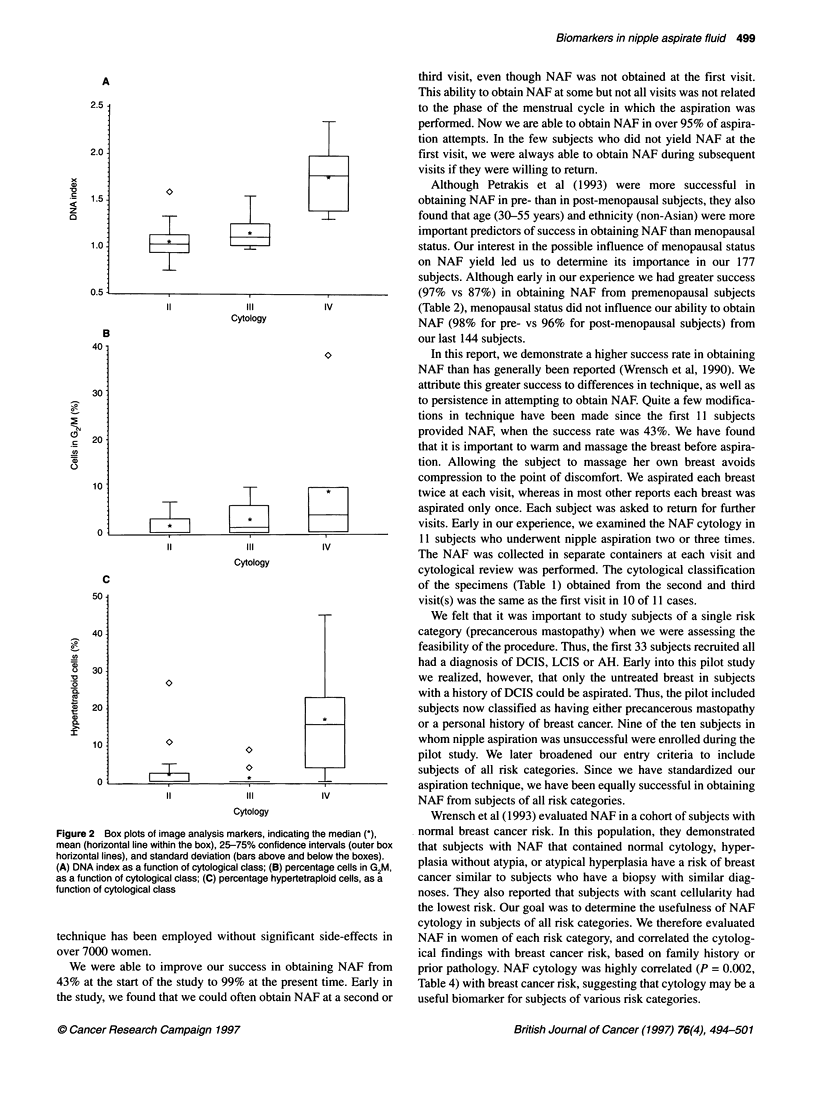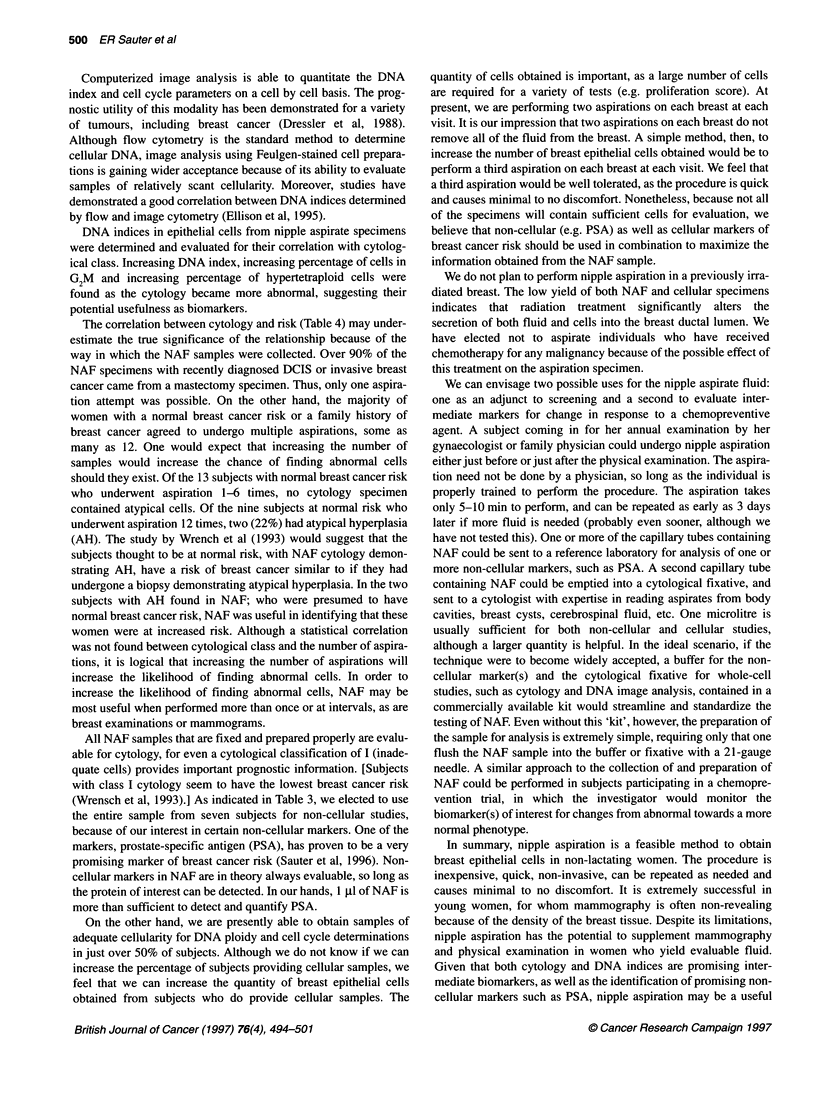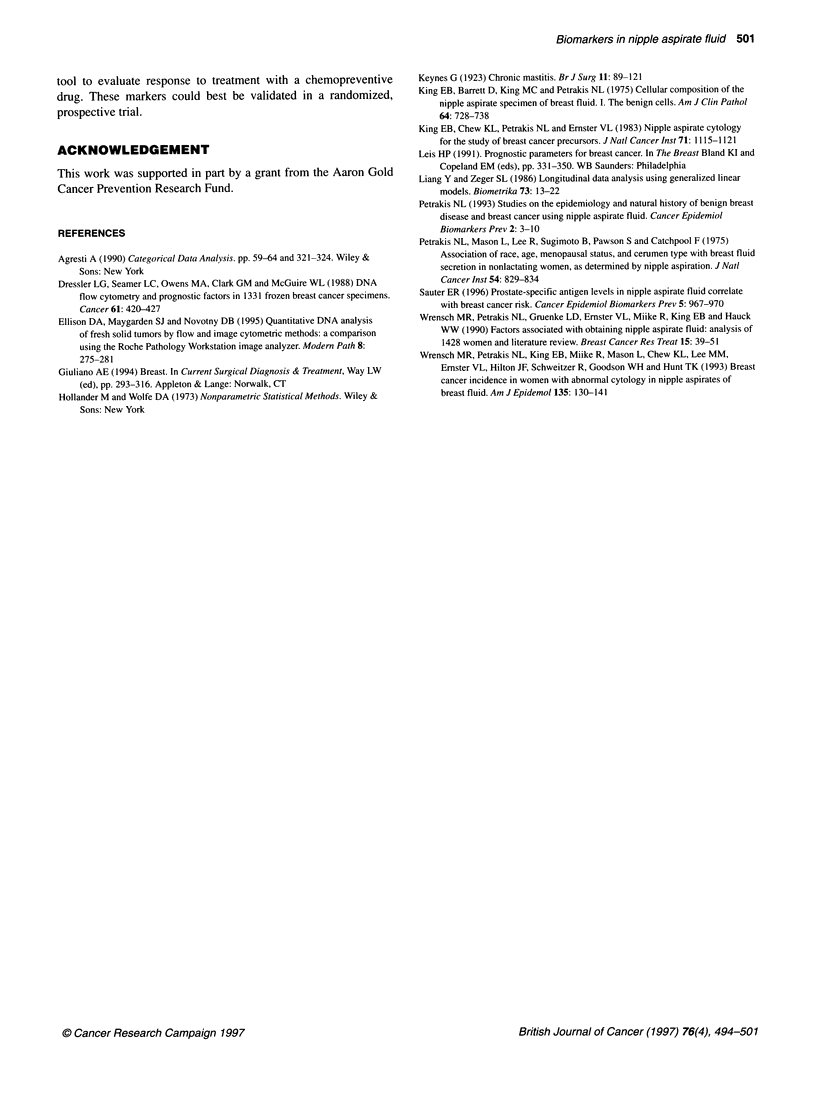# Nipple aspirate fluid: a promising non-invasive method to identify cellular markers of breast cancer risk.

**DOI:** 10.1038/bjc.1997.415

**Published:** 1997

**Authors:** E. R. Sauter, E. Ross, M. Daly, A. Klein-Szanto, P. F. Engstrom, A. Sorling, J. Malick, H. Ehya

**Affiliations:** Division of Population Science, Fox Chase Cancer Center, Philadelphia, PA 19111, USA.

## Abstract

**Images:**


British Journal of Cancer (1997) 76(4), 494–501
© 1997 Cancer Research Campaign

### Nipple aspirate fluid: a promising non-invasive method to identify cellular markers of breast cancer risk

**ER Sauter[1], E Ross[1], M Daly[1], A Klein-Szanto[2], PF Engstrom[1], A Sorling[2], J Malick[1] and H Ehya[2]**

The [1]Division of Population Science and the [2]Department of Pathology, Fox Chase Cancer Center, Philadelphia, PA 19111, USA

**Summary** To evaluate the feasibility of nipple aspiration and to identify intermediate markers of breast cancer risk, nipple aspirate fluid (NAF) was collected from 177 subjects using a modified breast pump. The first 33 subjects demonstrated that we could obtain NAF quickly, reliably and repeatedly. Specimens from the remaining 144 subjects were collected to evaluate promising cellular biomarkers. NAF was obtained in 167 out of 177 (94%) subjects overall and in 99% of the 144 most recent subjects. Sufficient NAF was obtained to evaluate cytology in 160 out of 167 (96%) cases and specimens were sufficiently cellular to analyse DNA markers in 53% of cases. Among the last 144 subjects, menopausal status did not influence the ability to obtain NAF. NAF cytology correlated with increased breast cancer risk ($P = 0.002$). Using computerized image analysis of NAF epithelial cells, DNA index ($P = 0.0002$), percentage of cells in $G_2M$ ($P = 0.05$) and percentage of cells with hypertetraploidy ($P = 0.002$) increased as cytology became more abnormal. Our data indicate that NAF can be obtained in essentially all eligible subjects; that breast epithelial cells are evaluable in > 95% of NAF samples for cytology and in over half of NAF samples for DNA index (ploidy) and cell cycle analysis; and that abnormal NAF cytology correlates with increased breast cancer risk. This suggests that biomarkers identified in nipple aspirate fluid may prove useful either as an adjunct to currently accepted breast cancer screening methods, or to evaluate response to a chemopreventive agent.

**Keywords:** nipple aspirate fluid; intermediate biomarker: breast cancer risk

Despite the death of 45 000 women annually in the United States from the disease, the ability to assess a woman's risk of developing breast cancer remains inadequate. The only well-established procedures to screen subjects for breast cancer are physical examination and mammography. Unfortunately, physical examination does not identify a significant number of early breast cancers and mammograms miss 10–40% of early breast cancers (Giuliano, 1994). Additional screening tools to identify precancer and early breast cancer are urgently needed.

Although the early detection of breast cancer will lead to a higher cure rate, the ideal form of treatment is prevention. The prevention of breast cancer is hindered by the difficulty in identifying an effective agent. Effective agents are difficult to identify in part because of the long period required for breast cancer to develop and, consequently, the requirement for lengthy clinical trials to test the efficacy of the agent, if the end point is the prevention of cancer. One way to shorten the time to finding of an effective agent is the identification of intermediate biomarkers, which are biological alterations in cells or tissue that occur between the time of initiation and tumour invasion. The theory is that an agent that partially or completely reverses the intermediate biomarker back to a normal phenotype may be interrupting carcinogenesis. Validation of the biomarker would require that the agent also decrease the incidence of cancer. Evaluating the effect of the agent requires the analysis of tissue, cells or non-cellular fluid. Nipple aspiration, which is simple, quick, reliable and repeatable, would be an excellent tool to evaluate the efficacy of a chemopreventive agent should intermediate biomarkers be identified in the fluid.

Present efforts to evaluate the breast directly, through evaluation of either tissue or individual cells, have been hindered because the analysis of these specimens generally requires an invasive procedure. The adult non-pregnant, non-lactating breast secretes fluid into the breast ductal system. This fluid can be obtained through aspiration of the nipple with a modified breast pump. Refinements in the ability to obtain this fluid, as well as epidemiological studies to identify subjects most likely to yield NAF, have been on-going for over 20 years. Nipple aspiration has the attractiveness of quickly, painlessly, and non-invasively obtaining breast epithelial cells, the cells at risk for transformation to breast cancer. Nonetheless, limitations in the ability to obtain NAF in 30–50% of subjects has hindered the development of the technique as a tool for both breast cancer screening and evaluating response to treatment (Petrakis, 1993).

Our study had two goals. The first was to determine if the procedure was feasible. That is, could NAF be obtained consistently and were the NAF samples sufficiently cellular such that accepted markers of prognosis in breast tumours (e.g. cytology and ploidy) could be evaluated in the fluid? If it could be demonstrated that the procedure was feasible, the next goal was to identify intermediate biomarkers in the NAF that would correlate with breast cancer risk. Subjects of all risk categories were recruited. Cytological study of the Papanicolaou-stained smears and computerized image analysis were used to identify promising cellular markers of breast cancer risk. We evaluated markers such as cellular atypia, DNA index or ploidy and S-phase fraction, which in breast carcinoma tissue specimens are known to correlate with prognosis

*Received 10 May 1996*
*Revised 24 January 1997*
*Accepted 17 February 1997*

*Correspondence to:* ER Sauter

(Leis, 1991). We hypothesized that higher levels of these markers in NAF epithelial cells would correlate with increasing breast cancer risk. Because of a previous report indicating that NAF was more successful in premenopausal subjects, we also evaluated the influence of menopausal status on our ability to obtain NAF. Our findings form the basis of this report.

### MATERIALS AND METHODS

### Subjects

One hundred and seventy-seven non-Asian subjects aged 30–65 years (women undergoing mastectomy excepted) were recruited between August 1994 and June 1996 after approval of the Fox Chase Institutional Review Board. Asians, as well as women under 30 or greater than 65 years, were excluded because the success in obtaining NAF from the intact breast in these groups is known to be low (Petrakis, 1993). These subjects were categorized by their risk for breast cancer as having no risk factors, a first-degree relative with breast cancer, a history of curative treatment for ductal carcinoma in situ (DCIS) or invasive breast cancer, precancerous mastopathy [atypical hyperplasia (AH) or lobular carcinoma in situ (LCIS) or recently diagnosed DCIS] or recently diagnosed invasive cancer of the breast. We relaxed the age limit for women undergoing mastectomy to include subjects up to 72 years old because: (1) there are no data outlining the NAF yield from a breast that has just been removed and (2) our preliminary attempts to obtain NAF in mastectomy specimens from women up to 72 years of age were successful. Aspiration visits spaced either 3 days apart (subjects undergoing 6–12 aspirations) or 2 weeks apart (subjects undergoing two or three aspirations) were performed by a trained physician or nurse clinician.

### Aspiration technique

After informed consent was obtained, nipple fluid was aspirated using a modified breast pump. The device consists of a 10-ml syringe attached to the end of a no. 4 endotracheal tube over which is placed a respiratory humidification adaptor. Each of these pieces is inexpensive and readily available in any hospital where mechanical respiratory support is provided.

For subjects in whom the intact breast was aspirated, the individual was seated in a comfortable position and the breast nipple cleansed with alcohol. After the alcohol evaporated, a warm, moist cloth was placed on each breast. After 1–2 min, the cloths were removed, the patient massaged her breast with both hands, and the plunger of the syringe was withdrawn to the 7-ml level and held for 15 s or until the patient experienced discomfort. A similar degree of suction was created with the syringe in mastectomy specimens obtained immediately after surgical excision. Fluid in the form of droplets was collected in capillary tubes. The quantity of fluid varied from 1 μl to 200 μl. The negative pressure produced was well tolerated. The procedure was repeated once on the same breast. Aspiration was then performed on the opposite breast, if present. If there was insufficient fluid from either breast to collect into one or more capillary tubes, or if the only breast that could be sampled (because of previous mastectomy) yielded no collected fluid, the patient was designated a non-secretor.

Occasionally, keratin plugs rather than NAF were obtained after suction was completed. The plugs were removed with an alcohol swab and suctioning repeated. Occasionally, suctioning was performed two or three times to remove all of the plugs. Fluid was then obtained frequently. In order to obtain additional fluid, the nipple was gently compressed by the subject between her fingers. One or two additional droplets of fluid often appeared.

### Cytology

#### *Specimen preparation*

The NAF was collected in 50-μl capillary tubes, rinsed into a container with 1 ml of 2% polyethylene glycol in ethanol–isopropanol and transported to the cytology laboratory for processing. In subjects with normal breast cancer risk, a family history of breast cancer, AH, or LCIS the samples were combined, given that the risk of invasive breast cancer is approximately equal in each breast. To evaluate the validity of this approach, early in our experience we collected NAF from nine subjects with AH or LCIS. The fluid from each breast was placed in separate containers. In seven out of nine cases, the cytology in both breasts was the same. In one of the remaining subjects, the abnormal NAF cytology was found in the breast opposite to where a prior biopsy had demonstrated AH, whereas in the second case the abnormal cytology was in the ipsilateral breast. If a cytology report was available from each breast, the report from the breast at highest risk was recorded. If a cytology report was available from each breast and the breasts were at equal risk, either the left or the right breast sample was selected randomly. If multiple cytological samples were analysed from the same breast, the most abnormal result was used in our analyses.

On the other hand, for subjects with previously treated DCIS or invasive breast cancer, only the breast without prior disease was aspirated. This was because the breast with disease had already been removed or radiated. For subjects with recently diagnosed but not yet definitively treated DCIS or invasive breast cancer, only the breast diagnosed with in situ or invasive disease was aspirated.

**Table 1** Cytological criteria for nipple aspirate specimens

*Scant mammary epithelial cells*
This includes acellular specimens, those with only foam cells or with fewer than ten mammary epithelial cells

*Benign mammary epithelial cells*
This consists of specimens containing more than ten mammary epithelial cells without cytological atypia. This category encompasses normal mammary cells, apocrine metaplasia, and duct hyperplasia without atypia. We subdivide this category into:
(a) Normal/non-papillary – single cells or small, loosely cohesive aggregates of cells.
(b) Hyperplastic – large three-dimensional clusters of cells (indicative of duct hyperplasia or papillomatosis)

*Atypical mammary epithelial cells*
This category consists of specimens with ten or more mammary epithelial cells that exhibit atypical features. Atypia is defined as nuclear enlargement and/or irregularity, increased nuclear to cytoplasmic ratio or a chromatin distribution abnormality short of obvious malignant criteria. It is noted whether the atypical cells are single or in papillary clusters.

*Malignant cells present*
This category consists of specimens that contain cells with unequivocal criteria of malignancy. If the malignant cells are fewer than ten, the specimens are designated as 'scanty evidence'

© Cancer Research Campaign 1997

**Figure 1** Photomicrographs of cytological preparations (Papanicolaou stain, ×1000). (**A**) Foam cells, a frequent constituent of NAF; (**B**) papillary cluster of benign epithelial cells without atypia; (**C**) papillary cluster of atypical mammary epithelial cells; (**D**) malignant epithelial cells

In each case, the entire specimen was cytocentrifuged onto ten glass slides. Three of the slides were used for cytological examination. If the slides contained less than ten epithelial cells, two additional slides were examined. The remaining slides (five or seven) were stored for biomarker studies. The slides selected for cytological examination were washed twice in 95% ethanol for 5 min each, rehydrated in tap water and stained by the Papanicolaou method.

### *Specimen interpretation*

The Papanicolaou-stained smears were examined by a cytopathologist experienced in breast cytology. Each specimen was designated (Table 1) as containing scant, benign, atypical or malignant cells, using a classification modified from King et al (1983). Representative photographs of various types of cells found in NAF, including benign, atypical and malignant epithelial cells, as well as foam cells, are illustrated in Figure 1.

### Image analysis

Only specimens with adequate cellularity (cytology classes II–IV) were evaluated by image analysis. Although 88 specimens (53%) were sufficiently cellular to perform image analysis, this technique was not employed until more than half of the subjects had been accrued. Thus far, 48 specimens have been evaluated.

### *Specimen preparation*

A standardized quantitative DNA staining kit (RIAS Feulgen Stain Kit, Roche Image Analysis Systems, Elon College, NC, USA) was used following the manufacturer's instructions. In brief, after rehydration the slides were processed for hydrolysis with 5 N hydrochloric acid for 60 min and then transferred to the staining solution (Schiff's reagent) for 1 h, rinsed, dehydrated and mounted with synthetic resin.

### *Specimen interpretation*

The Roche Pathology Workstation (Ellison et al, 1995) was used to evaluate nuclear ploidy, S-phase fraction, percentage of cells in $G_2M$ and percentage hypertetraploid cells (cells with more than twice their complement of DNA), using computerized image analysis of cytocentrifuged nipple aspirate epithelial cells stained with a standard Feulgen preparation. Human lymphocytes were used as a control diploid cell population. All epithelial cells if

© Cancer Research Campaign 1997

**Table 2** Yield of fluid and epithelial cells by menopausal status

| Status | Fluid obtained (%) | | Cytology[a] | | | |
|---|---|---|---|---|---|---|
| | Subjects | Attempts[b] | Cellular | Low cellularity | No fluid | Non-cellular studies only |
| Overall | | | | | | |
| Premenopausal | 83/86 (97) | 221/228 (97) | 45 | 35 | 3 | 3 |
| Post-menopausal | 84/91 (92) | 127/146 (87) | 43 | 37 | 7 | 4 |
| Totals | 167/177 (94) | 348/374 (93) | 88 | 72 | 10 | 7 |
| Last 144 subjects | | | | | | |
| Premenopausal | 70/71 (99) | 198/200 (98) | 38 | 29 | 1 | 3 |
| Post-menopausal | 73/73 (100) | 107/111 (96) | 40 | 29 | 0 | 4 |
| Totals | 143/144 (99) | 305/311 (98) | 78 | 58 | 1 | 7 |

[a]Cytology: if more than one cytological specimen was collected on a given subject, the most cellular specimen was counted; [b]Attempts: each subject may have undergone up to 12 aspiration attempts.

**Table 3** Yield of fluid and epithelial cells by breast cancer risk category

| Risk | Fluid obtained (%) | | Cytology[a] | | | |
|---|---|---|---|---|---|---|
| | Subjects | Attempts[b] | Cellular | Low cellularity | No fluid | Non-cellular studies only |
| Normal risk or family history | 48/49 (98) | 217/220 (99) | 19 | 29 | 1 | 0 |
| Personal history of breast cancer[c] | 14/16 (88) | 18/30 (84) | 3 | 8 | 2 | 3 |
| Precancerous mastopathy[c] | 32/39 (82) | 40/51 (75) | 22 | 10 | 7 | 0 |
| Invasive cancer | 73/73 (100) | 73/73 (100) | 44 | 25 | 0 | 4 |
| Totals | 167/177 (99) | 348/374 (95) | 88 | 72 | 10 | 7 |

[a]Cytology: a cellular specimen contains ≥ 10 breast epithelial cells on a slide. If more than one cytology specimen collected on a given subject, the more cellular specimen was counted; [b]attempts: each subject may have undergone one, two or three aspiration attempts; [c]personal history, precancerous mastopathy: the nine subjects in whom fluid was not obtained were enrolled very early in the study, before the standardization of our aspiration technique.

**Table 4** Cytology in nipple aspirate fluid specimens by risk category

| Risk | *n* | Cytological class | | | | *P*-value[a] |
|---|---|---|---|---|---|---|
| | | I | II | III | IV | |
| Normal risk or family history (FH) | 50 | 29 | 15 | 6 | 0 | |
| Personal history of breast cancer | 10 | 8 | 2 | 0 | 0 | NS |
| Precancerous mastopathy (PM) | 31 | 10 | 11 | 8 | 2 | 0.005 |
| Invasive cancer (IC) | 69 | 25 | 26 | 11 | 7 | 0.004 |
| Overall | | | | | | 0.002 |

[a]*P*-value: NS, not significant; 0.005, PM vs normal risk or family history of breast cancer; 0.004, IC vs normal risk or family history of breast cancer; 0.002, overall difference between the groups.

under 100 were present on a slide or a minimum of 100 cells if more were present were measured per case and type of stain. The average number of cells counted per specimen was 30.

### Statistical analysis

Cumulative logistic regression models (Agresti, 1990) were fitted to the data to determine whether the distribution of cytological groupings was associated with the percentage of cells in $G_2M$, percentage of cells in S-phase, DNA index, current use of hormone replacement therapy, current use of oral contraceptives, previous pregnancy, number of livebirths, number of miscarriages and whether the subject had difficulty becoming pregnant. Similarly, these methods were used to determine whether increasing breast cancer risk categories were associated with cytological grouping, percentage of hypertetraploid cells, percentage of cells in $G_2M$, percentage of cells in S-phase and ploidy. The data did not support the proportional odds assumption for the relationship between cytological group and percentage hypertetraploid cells. Therefore, the Kruskal–Wallis k-sample procedure (Hollander and Wolfe, 1973) was used to test the null hypothesis that individuals in the different cytological groups had identical distributions of hypertetraploid cells in the nipple aspirate fluid. Failure to reject the null hypothesis ($P > 0.05$) indicates that subgroups could not be distinguished. Rejection of the null hypothesis ($P < 0.05$) indicates that there are at least two subgroups where the hypertetraploid values are not identically distributed.

Fisher's exact two-sided procedures (Agresti, 1990) were used to determine whether a subject's ability to successfully provide NAF was associated with menopausal status. Logistic regression models were fitted to the data using generalized estimating equations methods (Liang and Zeger, 1986), to determine whether the menopausal status of the subject was associated with the probability of successfully obtaining NAF at any given attempt. To account for any potential within-subject correlation in the outcome variable, the test statistics were computed using the robust variance estimates.

© Cancer Research Campaign 1997 

**Table 5** Cellular markers in nipple aspirate fluid specimens by cytological class

| Cytology | Sample Size | DNA Index | | | $G_2M$ | | | Hypertetraploid cells (%) | | |
|---|---|---|---|---|---|---|---|---|---|---|
| | | Mean | Median | Range | Mean | Median | Range | Mean | Median | Range |
| No atypia | 28 | 1.06 | 1.03 | 0.74–1.59 | 1.61 | 0 | 0–6.67 | 2.14 | 0 | 0–26.79 |
| Atypical hyperplasia | 14 | 1.16 | 1.11 | 0.98–1.55 | 2.95 | 1.14 | 0–9.8 | 0.89 | 0 | 0–8.6 |
| Malignant | 6 | 1.77 | 1.78 | 1.31–2.35 | 9.20 | 3.85 | 0–37.8 | 17.29 | 15.79 | 0–45.16 |
| *P*-value | | | | 0.0002 | | 0.05 | | | 0.002 | |

Because image analysis has only recently been employed, 48 specimens have been evaluated in both the DNA index and % hypertetraploid categories.

### RESULTS

The correlation of breast cancer risk and NAF cytology with a variety of clinical factors, including current use of birth control pills (BCP), current use of hormone replacement therapy (HRT), whether the subject was ever pregnant, the number of livebirths, the number of miscarriages, the subject's difficulty becoming pregnant, the phase of the menstrual cycle in which the sample was obtained and the ease with which NAF was obtained were evaluated. None of these factors correlated with breast cancer risk or NAF cytology, although the power of the analysis of current use of birth control pills (BCP) and current use of hormone replacement therapy (HRT) was diminished by having an unbalanced population (few subjects were currently using either BCP or HRT). None of the clinical factors analysed affected the ability to obtain NAF. Nor did the interval between aspirations or the number of aspirations affect the ability to obtain fluid or the volume of NAF obtained on a given visit. Our cytologist did not identify blood in any NAF sample from women with an intact breast and rarely found blood in NAF from mastectomy specimens. Using image analysis, the amount of DNA in the breast epithelial cells obtained from NAF and the percentage of cells in each phase of the cell cycle were also evaluated. Although ploidy, the percentage of hypertetraploid cells and the percentage of cells in $G_2M$ did correlate with NAF cytology, the percentage of cells in the S-phase of the cell cycle did not.

To evaluate the influence of menopausal status on our ability to obtain NAF, our success in premenopausal vs post-menopausal subjects was compared (Table 2). The data were presented in two ways: first as the entire experience with 177 subjects and then with our most recent 144 subjects, which reflects our success with nipple aspiration once the technique was standardized. We had greater overall success per aspiration in pre- vs post-menopausal subjects (97% vs 87%, $P < 0.001$), although this difference was not seen in the last 144 subjects (98% vs 96%, *P* not significant).

Early in the study, we also assessed the effect of breast irradiation on our ability to obtain NAF specimens. Four subjects with DCIS who underwent prior radiation had their radiated breast aspirated twice or three times for a total of 11 aspirations. NAF was obtained only twice and both samples had scant cellularity.

Our success in obtaining both NAF and cellular specimens in subjects classified by breast cancer risk is outlined in Table 3. NAF was obtained in 167 out of 177 subjects in 348 out of 374 attempts. Fifty-three per cent (88 out of 167) of the specimens contained ten or more breast epithelial cells on a slide, the criterion used to carry out cellular biomarker determinations.

The correlation of nipple aspirate cytology and image analysis parameters (DNA index, percentage of hypertetraploid cells, S-phase fraction, and percentage of cells in $G_2M$) with risk category was next evaluated (Table 4). Risk was classified as normal risk or family history of breast cancer, personal history of breast cancer (prior DCIS or invasive cancer), precancerous mastopathy or invasive cancer. Overall, nipple aspirate cytology ($P = 0.002$) was significantly associated with breast cancer risk. Each increased risk category was compared with normal risk/family history of breast cancer. Pairwise comparisons revealed that samples from individuals with invasive cancer ($P = 0.004$) or precancerous mastopathy ($P = 0.005$) were significantly more likely to contain more abnormal cells.

Cellular markers, including DNA index, percentage of cells in the S-phase fraction, percentage of cells in $G_2M$ of the cell cycle and the percentage cells with more than twice the normal amount of DNA (percentage hypertetraploid cells) were evaluated using image analysis (Tables). DNA index ($P = 0.0002$), the percentage of cells in $G_2M$ ($P = 0.05$) and the percentage of hypertetraploid cells ($P = 0.002$) increased as cytology became more abnormal (Figure 2). Although there was a gradual increase in the DNA index and in the percentage of cells in $G_2M$ of the cell cycle that gradually increased as the cytology became more abnormal, the pattern seen for cells with hypertetraploidy was slightly different. In the latter case, the percentage of hypertetraploid cells was similar for cytological classes II and III, whereas there was a sudden increase in the number of hypertetraploid cells with class IV (malignant) cytology.

### DISCUSSION

The breast ducts of adult non-pregnant women secrete small amounts of fluid (Keynes, 1923). This fluid does not escape because the nipple ducts are occluded by smooth muscle contraction, dried secretions and keratinized epithelium. Breast fluid can be obtained by nipple aspiration in a significant proportion of women without spontanous nipple discharge with the use of a modified breast pump (Petrakis et al, 1975). This fluid contains several types of cells, including exfoliated breast epithelial cells (King et al, 1975). Because breast cancer develops from ductal and lobular epithelium, NAF is a potentially useful epidemiological and clinical research tool. A major limitation of the technique has been the lack of ability to obtain NAF in all women, and when fluid was obtained it frequently contained few or no breast epithelial cells.

Published data from Petrakis et al (1993) indicate that the highest yield of NAF is from women aged 30–55 years with early onset of menarche, non-Asian ethnicity and prior parity and/or lacation. This procedure has yielded NAF in over 50% of premenopausal Caucasian and African–American women over the age of 30. With a definition of ≥ 10 epithelial cells as an adequate aspiration, others have obtained an adequate specimen in 42% of women aspirated (King et al, 1983). The nipple aspiration

 © Cancer Research Campaign 1997

**Figure 2** Box plots of image analysis markers, indicating the median (*), mean (horizontal line within the box), 25–75% confidence intervals (outer box horizontal lines), and standard deviation (bars above and below the boxes). (**A**) DNA index as a function of cytological class; (**B**) percentage cells in $G_2M$, as a function of cytological class; (**C**) percentage hypertetraploid cells, as a function of cytological class technique has been employed without significant side-effects in over 7000 women.

We were able to improve our success in obtaining NAF from 43% at the start of the study to 99% at the present time. Early in the study, we found that we could often obtain NAF at a second or third visit, even though NAF was not obtained at the first visit. This ability to obtain NAF at some but not all visits was not related to the phase of the menstrual cycle in which the aspiration was performed. Now we are able to obtain NAF in over 95% of aspiration attempts. In the few subjects who did not yield NAF at the first visit, we were always able to obtain NAF during subsequent visits if they were willing to return.

Although Petrakis et al (1993) were more successful in obtaining NAF in pre- than in post-menopausal subjects, they also found that age (30–55 years) and ethnicity (non-Asian) were more important predictors of success in obtaining NAF than menopausal status. Our interest in the possible influence of menopausal status on NAF yield led us to determine its importance in our 177 subjects. Although early in our experience we had greater success (97% vs 87%) in obtaining NAF from premenopausal subjects (Table 2), menopausal status did not influence our ability to obtain NAF (98% for pre- vs 96% for post-menopausal subjects) from our last 144 subjects.

In this report, we demonstrate a higher success rate in obtaining NAF than has generally been reported (Wrensch et al, 1990). We attribute this greater success to differences in technique, as well as to persistence in attempting to obtain NAF. Quite a few modifications in technique have been made since the first 11 subjects provided NAF, when the success rate was 43%. We have found that it is important to warm and massage the breast before aspiration. Allowing the subject to massage her own breast avoids compression to the point of discomfort. We aspirated each breast twice at each visit, whereas in most other reports each breast was aspirated only once. Each subject was asked to return for further visits. Early in our experience, we examined the NAF cytology in 11 subjects who underwent nipple aspiration two or three times. The NAF was collected in separate containers at each visit and cytological review was performed. The cytological classification of the specimens (Table 1) obtained from the second and third visit(s) was the same as the first visit in 10 of 11 cases.

We felt that it was important to study subjects of a single risk category (precancerous mastopathy) when we were assessing the feasibility of the procedure. Thus, the first 33 subjects recruited all had a diagnosis of DCIS, LCIS or AH. Early into this pilot study we realized, however, that only the untreated breast in subjects with a history of DCIS could be aspirated. Thus, the pilot included subjects now classified as having either precancerous mastopathy or a personal history of breast cancer. Nine of the ten subjects in whom nipple aspiration was unsuccessful were enrolled during the pilot study. We later broadened our entry criteria to include subjects of all risk categories. Since we have standardized our aspiration technique, we have been equally successful in obtaining NAF from subjects of all risk categories.

Wrensch et al (1993) evaluated NAF in a cohort of subjects with normal breast cancer risk. In this population, they demonstrated that subjects with NAF that contained normal cytology, hyperplasia without atypia, or atypical hyperplasia have a risk of breast cancer similar to subjects who have a biopsy with similar diagnoses. They also reported that subjects with scant cellularity had the lowest risk. Our goal was to determine the usefulness of NAF cytology in subjects of all risk categories. We therefore evaluated NAF in women of each risk category, and correlated the cytological findings with breast cancer risk, based on family history or prior pathology. NAF cytology was highly correlated ($P$ = 0.002, Table 4) with breast cancer risk, suggesting that cytology may be a useful biomarker for subjects of various risk categories.

© Cancer Research Campaign 1997

Computerized image analysis is able to quantitate the DNA index and cell cycle parameters on a cell by cell basis. The prognostic utility of this modality has been demonstrated for a variety of tumours, including breast cancer (Dressler et al, 1988). Although flow cytometry is the standard method to determine cellular DNA, image analysis using Feulgen-stained cell preparations is gaining wider acceptance because of its ability to evaluate samples of relatively scant cellularity. Moreover, studies have demonstrated a good correlation between DNA indices determined by flow and image cytometry (Ellison et al, 1995).

DNA indices in epithelial cells from nipple aspirate specimens were determined and evaluated for their correlation with cytological class. Increasing DNA index, increasing percentage of cells in $G_2M$ and increasing percentage of hypertetraploid cells were found as the cytology became more abnormal, suggesting their potential usefulness as biomarkers.

The correlation between cytology and risk (Table 4) may underestimate the true significance of the relationship because of the way in which the NAF samples were collected. Over 90% of the NAF specimens with recently diagnosed DCIS or invasive breast cancer came from a mastectomy specimen. Thus, only one aspiration attempt was possible. On the other hand, the majority of women with a normal breast cancer risk or a family history of breast cancer agreed to undergo multiple aspirations, some as many as 12. One would expect that increasing the number of samples would increase the chance of finding abnormal cells should they exist. Of the 13 subjects with normal breast cancer risk who underwent aspiration 1–6 times, no cytology specimen contained atypical cells. Of the nine subjects at normal risk who underwent aspiration 12 times, two (22%) had atypical hyperplasia (AH). The study by Wrench et al (1993) would suggest that the subjects thought to be at normal risk, with NAF cytology demonstrating AH, have a risk of breast cancer similar to if they had undergone a biopsy demonstrating atypical hyperplasia. In the two subjects with AH found in NAF; who were presumed to have normal breast cancer risk, NAF was useful in identifying that these women were at increased risk. Although a statistical correlation was not found between cytological class and the number of aspirations, it is logical that increasing the number of aspirations will increase the likelihood of finding abnormal cells. In order to increase the likelihood of finding abnormal cells, NAF may be most useful when performed more than once or at intervals, as are breast examinations or mammograms.

All NAF samples that are fixed and prepared properly are evaluable for cytology, for even a cytological classification of I (inadequate cells) provides important prognostic information. [Subjects with class I cytology seem to have the lowest breast cancer risk (Wrensch et al, 1993).] As indicated in Table 3, we elected to use the entire sample from seven subjects for non-cellular studies, because of our interest in certain non-cellular markers. One of the markers, prostate-specific antigen (PSA), has proven to be a very promising marker of breast cancer risk (Sauter et al, 1996). Non-cellular markers in NAF are in theory always evaluable, so long as the protein of interest can be detected. In our hands, 1 μl of NAF is more than sufficient to detect and quantify PSA.

On the other hand, we are presently able to obtain samples of adequate cellularity for DNA ploidy and cell cycle determinations in just over 50% of subjects. Although we do not know if we can increase the percentage of subjects providing cellular samples, we feel that we can increase the quantity of breast epithelial cells obtained from subjects who do provide cellular samples. The quantity of cells obtained is important, as a large number of cells are required for a variety of tests (e.g. proliferation score). At present, we are performing two aspirations on each breast at each visit. It is our impression that two aspirations on each breast do not remove all of the fluid from the breast. A simple method, then, to increase the number of breast epithelial cells obtained would be to perform a third aspiration on each breast at each visit. We feel that a third aspiration would be well tolerated, as the procedure is quick and causes minimal to no discomfort. Nonetheless, because not all of the specimens will contain sufficient cells for evaluation, we believe that non-cellular (e.g. PSA) as well as cellular markers of breast cancer risk should be used in combination to maximize the information obtained from the NAF sample.

We do not plan to perform nipple aspiration in a previously irradiated breast. The low yield of both NAF and cellular specimens indicates that radiation treatment significantly alters the secretion of both fluid and cells into the breast ductal lumen. We have elected not to aspirate individuals who have received chemotherapy for any malignancy because of the possible effect of this treatment on the aspiration specimen.

We can envisage two possible uses for the nipple aspirate fluid: one as an adjunct to screening and a second to evaluate intermediate markers for change in response to a chemopreventive agent. A subject coming in for her annual examination by her gynaecologist or family physician could undergo nipple aspiration either just before or just after the physical examination. The aspiration need not be done by a physician, so long as the individual is properly trained to perform the procedure. The aspiration takes only 5–10 min to perform, and can be repeated as early as 3 days later if more fluid is needed (probably even sooner, although we have not tested this). One or more of the capillary tubes containing NAF could be sent to a reference laboratory for analysis of one or more non-cellular markers, such as PSA. A second capillary tube containing NAF could be emptied into a cytological fixative, and sent to a cytologist with expertise in reading aspirates from body cavities, breast cysts, cerebrospinal fluid, etc. One microlitre is usually sufficient for both non-cellular and cellular studies, although a larger quantity is helpful. In the ideal scenario, if the technique were to become widely accepted, a buffer for the non-cellular marker(s) and the cytological fixative for whole-cell studies, such as cytology and DNA image analysis, contained in a commercially available kit would streamline and standardize the testing of NAF. Even without this 'kit', however, the preparation of the sample for analysis is extremely simple, requiring only that one flush the NAF sample into the buffer or fixative with a 21-gauge needle. A similar approach to the collection of and preparation of NAF could be performed in subjects participating in a chemoprevention trial, in which the investigator would monitor the biomarker(s) of interest for changes from abnormal towards a more normal phenotype.

In summary, nipple aspiration is a feasible method to obtain breast epithelial cells in non-lactating women. The procedure is inexpensive, quick, non-invasive, can be repeated as needed and causes minimal to no discomfort. It is extremely successful in young women, for whom mammography is often non-revealing because of the density of the breast tissue. Despite its limitations, nipple aspiration has the potential to supplement mammography and physical examination in women who yield evaluable fluid. Given that both cytology and DNA indices are promising intermediate biomarkers, as well as the identification of promising non-cellular markers such as PSA, nipple aspiration may be a useful

© Cancer Research Campaign 1997

tool to evaluate response to treatment with a chemopreventive drug. These markers could best be validated in a randomized, prospective trial.

### ACKNOWLEDGEMENT

This work was supported in part by a grant from the Aaron Gold Cancer Prevention Research Fund.

### REFERENCES

Agresti A (1990) *Categorical Data Analysis*. pp. 59–64 and 321–324. Wiley & Sons: New York

Dressler LG, Seamer LC, Owens MA, Clark GM and McGuire WL (1988) DNA flow cytometry and prognostic factors in 1331 frozen breast cancer specimens. *Cancer* **61**: 420–427

Ellison DA, Maygarden SJ and Novotny DB (1995) Quantitative DNA analysis of fresh solid tumors by flow and image cytometric methods: a comparison using the Roche Pathology Workstation image analyzer. *Modern Path* **8**: 275–281

Giuliano AE (1994) Breast. In *Current Surgical Diagnosis & Treatment*, Way LW (ed), pp. 293–316. Appleton & Lange: Norwalk, CT

Hollander M and Wolfe DA (1973) *Nonparametric Statistical Methods*. Wiley & Sons: New York

Keynes G (1923) Chronic mastitis. *Br J Surg* **11**: 89–121

King EB, Barrett D, King MC and Petrakis NL (1975) Cellular composition of the nipple aspirate specimen of breast fluid. I. The benign cells. *Am J Clin Pathol* **64**: 728–738

King EB, Chew KL, Petrakis NL and Ernster VL (1983) Nipple aspirate cytology for the study of breast cancer precursors. *J Natl Cancer Inst* **71**: 1115–1121

Leis HP (1991). Prognostic parameters for breast cancer. In *The Breast* Bland KI and Copeland EM (eds), pp. 331–350. WB Saunders: Philadelphia

Liang Y and Zeger SL (1986) Longitudinal data analysis using generalized linear models. *Biometrika* **73**: 13–22

Petrakis NL (1993) Studies on the epidemiology and natural history of benign breast disease and breast cancer using nipple aspirate fluid. *Cancer Epidemiol Biomarkers Prev* **2**: 3–10

Petrakis NL, Mason L, Lee R, Sugimoto B, Pawson S and Catchpool F (1975) Association of race, age, menopausal status, and cerumen type with breast fluid secretion in nonlactating women, as determined by nipple aspiration. *J Natl Cancer Inst* **54**: 829–834

Sauter ER (1996) Prostate-specific antigen levels in nipple aspirate fluid correlate with breast cancer risk. *Cancer Epidemiol Biomarkers Prev* **5**: 967–970

Wrensch MR, Petrakis NL, Gruenke LD, Ernster VL, Miike R, King EB and Hauck WW (1990) Factors associated with obtaining nipple aspirate fluid: analysis of 1428 women and literature review. *Breast Cancer Res Treat* **15**: 39–51

Wrensch MR, Petrakis NL, King EB, Miike R, Mason L, Chew KL, Lee MM, Ernster VL, Hilton JF, Schweitzer R, Goodson WH and Hunt TK (1993) Breast cancer incidence in women with abnormal cytology in nipple aspirates of breast fluid. *Am J Epidemol* **135**: 130–141

© Cancer Research Campaign 1997